# The phenomenal epigenome in neurodevelopmental disorders

**DOI:** 10.1093/hmg/ddaa175

**Published:** 2020-08-07

**Authors:** Ummi Ciptasari, Hans van Bokhoven

**Affiliations:** Department of Human Genetics, Donders Institute for Brain, Cognition and Behavior, Radboud university medical center, 6500 HB Nijmegen, The Netherlands; Department of Human Genetics, Donders Institute for Brain, Cognition and Behavior, Radboud university medical center, 6500 HB Nijmegen, The Netherlands; Department of Cognitive Neuroscience, Donders Institute for Brain, Cognition and Behavior, Radboud university medical center, 6500 HB Nijmegen, The Netherlands

## Abstract

Disruption of chromatin structure due to epimutations is a leading genetic etiology of neurodevelopmental disorders, collectively known as chromatinopathies. We show that there is an increasing level of convergence from the high diversity of genes that are affected by mutations to the molecular networks and pathways involving the respective proteins, the disrupted cellular and subcellular processes, and their consequence for higher order cellular network function. This convergence is ultimately reflected by specific phenotypic features shared across the various chromatinopathies. Based on these observations, we propose that the commonly disrupted molecular and cellular anomalies might provide a rational target for the development of symptomatic interventions for defined groups of genetically distinct neurodevelopmental disorders.

## Introduction

Almost all cells in our body contain the same DNA, yet clearly a large variety of different cell types can be distinguished by their morphology, function and RNA expression profiles (1). The differences between these cell types are controlled by chemical modifications and structural organization of the chromatin, popularly alluded to as ‘epigenetic mechanisms’. Epigenetic modifications guide the patterns of gene expression and are installed along pre-defined developmental lineages from the zygote to the full set of differentiated cells and organs (2). Such epigenetic modifications can be transmitted between successive generations of cells to maintain cell identity. In addition, epigenetic modifications can also be introduced by external triggers, e.g. exposure to toxins, nutrients and stress, which can have life-long effects. Consequently, epigenetics is considered a rational mediator of gene × environment interactions. Finally, epigenetic modifications are required for memory formation and other forms of learning, suggesting that epigenetic modifications might act as a cellular memory (3,4). In view of this, it is not surprising that disruptions of the epigenetic machinery are often underlying mental disorders alluded to as chromatinopathies (5).

## Epimutations in genes causing neurodevelopmental disorders

Our review is confined to monogenetic neurodevelopmental disorders (NDDs) caused by highly penetrant secondary epimutations causing cognitive impairment as the major and consistent feature. Previously, we and others have made compilations of such disorders and respective genes carrying epimutations (6–11). These overviews vary because of the rapid increase of new NDD genes. In addition, the definition ‘epigenetic genes’ is variably used in drafting these overviews. A useful dynamic overview is maintained by the group of H. Bjornsson at www.epigeneticmachinery.org (12).

For this review we have defined chromatinopathy genes based on their ability to chemically modify or remodel chromatin structure. This essentially encompasses a list of genes whose corresponding protein products have a catalytic function in installing post-translational histone modifications and DNA modifications (writers), removing such modifications (erasers) or have chromatin remodeling activity (remodelers). The readers of chromatin structures are confined to proteins that have a reader domain for binding to specific chromatin modifications and chromatin architectural proteins. To select these genes, we first identified all human genes that satisfied these criteria by querying the Gene Ontology (GO) database (http://amigo.geneontology.org/amigo/search/ontology; release May 2020) with the search term ‘chromatin organization’ (GO:0006325). Chromatin organization is defined as any process that results in the specification, formation or maintenance of the physical structure of eukaryotic chromatin and together with its child terms (Supplementary Material, Table S1) seems to cover all chromatin modulating processes. In total 771 of 19 858 human genes in the GO database are classified with the GO term ‘chromatin organization’. This group was complemented by the selection of 295 genes from the epigenetic machinery database, which harbors a wider collection of stringently selected chromatin readers, resulting in a collection of 872 genes (12) (Supplementary Material, Table S2). Next, we selected all genes for which causative variants have been reported to cause an NDD, using the SysID database (https://sysid.cmbi.umcn.nl/). SysID comprises 1333 verified NDD genes (March 2020) for which causative monogenic variants have been reported in multiple unrelated individuals with a neurodevelopmental phenotype (13). Overlay of the SysID genes with the chromatin control genes revealed 121 SysID genes with the GO annotation chromatin organization. In line with previous notions, this analysis shows that genes with an epigenetic function are enriched in the list of NDD genes compared to the list containing all human genes by a factor of 2.33 (*P* < 0.0001) (8,14), which is similar to the enrichment of synapse-related genes [1354 genes with GO term synapse, 210 in SysID; enrichment of 2,31 (*P* < 0.0001)].To be sure we did not miss a major number of genes implicated in NDDs, we visually inspected the SysID database, which revealed *ADNP*, *YY1* and *RBPJ* as additional genes. Furthermore, a query of other disease gene databases, such as the Developmental Disorders Genotype-to-Phenotype database (DDG2P) table from the ‘Deciphering Developmental Disorders’ project and the AutDB autism database (15,16), revealed an additional six high-confidence chromatin-related genes that were still ranked as candidates in SysID: *DDB2*, *DDX11*, *GPX4*, *HDAC4*, *KMT5B* and *MORC2*, bringing the total overlap to 130 genes (Supplementary Material, Table S3).

The 130 epigenetic NDD genes encompass the writers, erasers, remodelers and readers. Only 18 genes carry bi-allelic variants that cause an NDD phenotype. The majority is either X-linked or has disease-causing variants that manifest in a dominant pattern that often arise by *de novo* mutation. This indicates that chromatinopathy genes are highly dosage sensitive. Histone methyltransferases (HMTs) are particularly abundant amongst the writers, followed by histone acetyltransferases (HATs), and other rare chromatin modifiers (ubiquitination, phosphorylation, biotinylation, *O*-GlcNAcylation and DNA methylation). Members of the superfamily II helicases involved in adenosine triphosphate (ATP)-dependent nucleosome translocation are prominent in the group of remodelers. These helicases cover five conserved protein complexes: (1) SWI/SNF (Switch/Sucrose Non-Fermentable) [a.k.a. BRG1-associated factors (BAF)], (2) imitation switch, (3) chromatin helicase DNA binding (CHD), (4) INO80/SWR1 and (5) ATRX.

## Chromatinopathy genes cluster in epigenetic modules

The functional interactions across chromatinopathy genes provide a first step to picture how epigenetic dysregulation leads to neurodevelopmental defects. To uncover possible genotype–phenotype network associations, hierarchical clustering of functional interactions with the 130 genes was performed using STRING (17). Using stringent connectivity based on experimental evidence, several chromatinopathy genes are not clearly connected to others. However, abundant direct interactions between chromatinopathy genes catch the eye with major epigenetic regulator complexes such as complex of proteins associated with Set1 (COMPASS), polycomb recessive complex 2 (PRC2), nucleosome remodeling deacetylase (NuRD) and BAF complex as converging hubs in the network. These complexes are connected to one another by other chromatinopathy proteins, such as β-catenin (CTNNB1) and transcriptional activators/HAT p300 and cAMP response element-binding protein (CREB) binding protein (CREBBP/CBP).

**Figure 1:**
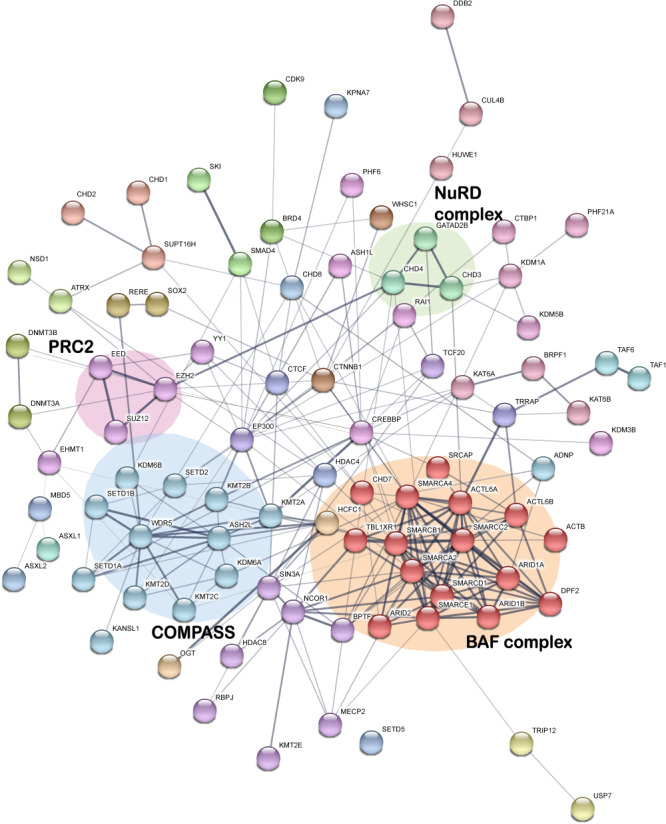
Simplified protein–protein interaction network of compiled chromatinopathy genes generated by StringDB (version 11.0, MCL clustering) analysis of chromatinopathy genes. For clarity, only proteins with interactions are shown. Connecting lines showing medium- and high-confidence experimental interactions (confidence > 0.400) with line thickness indicates the strength of data support.

The BAF complex, or the mammalian SWI/SNF complex, emerged as the strongest hub across chromatinopathy proteins (18). The complex has the ability to mobilize nucleosomes and is composed by core ATPase proteins and unique components dependent on the cell type in which they function. BAF complexes seem to have a genome-wide control on both active H3K4me2 and repressive H3K27me3 marks during late cortical development by directly interacting with the corresponding demethylases (19). Thus, inactivation of BAF complexes leads to H3K27me3-linked silencing (via KDM6A/B) of neuronal differentiation-related genes with concurrent H3K4me2-mediated activation (via KDM1A) of proliferation-associated genes via derepression of WNT signaling. Mutations in genes encoding various subunits of BAF complex often lead to what is known as SWI/SNF-related intellectual disability (ID) or BAFopathies, including Coffin–Siris syndrome (CSS), Nicolaides–Baraitser syndrome and other ID disorders, which share phenotypic features of developmental delay, ID, coarse facial features and phalangeal abnormalities (20,21). The BAFopathies represent a great example of the genetic principle that mutation of components of the same protein complex or nodes of the same molecular pathway give rise to disorders with overlapping phenotypes. A similar genotype–phenotype network association has been identified for Kleefstra syndrome, which is caused by loss-of-function mutations of *EHMT1*, encoding euchromatic histone methyltransferase 1 (EHMT1), a transcriptional repressor via mono- and dimethylation of histone 3 lysine 9 (H3K9me1/2). Exome sequencing of individuals with a phenotype strongly reminiscent of Kleefstra syndrome (Kleefstra syndrome spectrum [KSS]) revealed *de novo* mutations in several other chromatinopathy genes, including *MBD5*, *KMT2C* and the BAF complex gene *SMARCB1* (22). The differential phenotypes of CSS and KSS associated with *SMARCB1* might be explained by the different functional effects of the respective variants in addition to the clinical variability typically observed for individual chromatinopathy disorders (21,23,24). Physical and functional interactions amongst the KSS proteins revealed an EHMT1-associated chromatin-modification module, which is connected to the BAF, COMPASS and PRC2 complexes (Fig. 1).

Polycomb recessive complex 1 (PRC1) and PRC2 are both responsible for silencing genes unnecessary for the current state of the cells through monoubiquitination of H2AK119 and methylation of H3K27, respectively. PRC2 is a highly conserved multimeric complex consisting of four core proteins: EZH1/2, EED, SUZ12 and RBBP4/7, and a variety of accessory proteins that co-regulate PRC2 activity (25). Pathogenic mutations in *EZH2*, *SUZ12* and *EED* are seen in overgrowth syndromes with variable ID, such as Weaver syndrome and Cohen–Gibson syndrome (26–28). PRC2 interacts with DNMT3A. Somatic DNMT3A dominant-negative/loss-of-function mutations that lead to DNA hypomethylation at PRC2-responsive loci are frequently seen in hematological disorders. Similar germline mutations are also encountered in Tatton–Brown–Rahman syndrome, which is characterized by overgrowth, ID, tall stature, distinctive facial appearance and in some cases leukemia (29). Interestingly, *DNMT3A* gain-of-function mutations in Heyn–Sproul–Jackson syndrome are associated with hypermethylation at PRC2 loci and pre- and postnatal growth retardation (30). Other connections of PRC2 to chromatinopathy proteins comprise NSD1, underlying another overgrowth disorder (Sotos syndrome), YY1 (Gabriele–de Vries syndrome) and ATRX (several X-linked ID disorders).

PRC2 cooperatively interacts also with EHMT1 to exert repressive activity by PRC2-installed H3K27me3 marks. The repression is countered by the H3K27 demethylase activity of KDM6A (31,32), which is affected by hemizygous loss-of-function mutations in Kabuki syndrome type 2 (33). KDM6A is an integral regulator of macromolecular complexes called COMPASS that consist of variable constellations of SET family HMT with H3K4me1/2/3 activity (SETD1A/B and MLL/KMT2 proteins) (34). Germline mutations in all COMPASS methyltransferases have been implicated in NDDs, which share a range of clinical phenotypes including prominent cognitive impairment, behavioral abnormalities and features of abnormal craniofacial, skeletal and cardiovascular development (22,35–37). Interestingly, KMT2D, which is mutated in Kabuki syndrome type 1, interacts with KDM6A and CBP/p300 HAT (Rubinstein–Taybi syndrome) to promote transcriptional activation (38,39). KMT2C was found to be mutated in a number of KSS patients, which is somewhat surprising as KMT2C and EHMT1 have opposing activities as transcriptional activator and repressor, respectively. Many other interactions between COMPASS and other chromatinopathy proteins have been uncovered, such as with the chromatin remodeler CHD8, which activates expression of BRG1-associated BAF complexes and recruits MLL/KMT2 COMPASS complexes to promote central nervous system myelination and myelin repair (18,40,41).

## Transcriptional convergence in neurogenesis: WNT signaling

Not surprisingly, epimutations in chromatinopathies are associated with transcriptional deregulation of genes that are critical for proper neurodevelopment as well as genes that are important for acute neurophysiological processes. Disruption of genes during neurogenesis is often associated with imbalances in proliferation and differentiation of neural progenitors cells (NPCs), giving rise to structural brain anomalies such as micro/macrocephaly and cortical malformations. Previous integrated analysis of six exome studies uncovered a network of genes mutated in autism spectrum disorder (ASD) and ID highlighting proteins converging on β-catenin as a part of the WNT signaling pathway (41). In support of this, a recent study defined molecular and functional subclasses within a set of 30 autism-related genes (in fact better defined as NDD genes) based on the *in vitro* neurogenic potency of pluripotent stem cells carrying engineered mutations in these genes (42). This identified a class of eight genes with mutations associated with increased proliferation and inhibited neurogenesis (class 1 comprising chromatinopathy genes *ASH1L*, *ASXL3 KDM5B* and *KMT2C*). The reduced neurogenesis was strongly correlated with a reduced response to WNT signaling. Notably, five of the eight class 1 genes are known as regulators of polycomb, which like WNT is critical for coordinating the balance between NPC proliferation and differentiation (43–48). PRC2 positively regulates WNT/β-catenin signaling in various cell types through the silencing of WNT antagonists, and *EZH2* expression maintains NPC proliferation and is negatively correlated with neuronal differentiation (49–51). Accordingly, mutations of *EZH2* in Weaver syndrome are associated with overgrowth and macrocephaly. It should be noted, however, that the study of Cederquist *et al*. (42) also uncovered a smaller class of genes with the opposite neurogenic effects, which did not have an altered WNT response despite the fact that two chromatinopathy genes from this class, *KMT2A* and *CHD8*, are also regulators of polycomb. Moreover, mutations in these genes have variable growth defects in patients. This observation reinforces observations that the neurogenic properties of WNT and polycomb complex might be dependent on the specifics of the progenitor cells and the timing in cortical development (48).

Nevertheless, these exciting data reinforce the notion that the balance between proliferation and differentiation controlled by WNT signaling is mirrored by a delicate chromatin-based balance between activation and repression involving components of PRC2 and COMPASS. Previously, also the BAF complex was shown to control WNT signaling. Loss-of-function mutations affecting individual components in BAFopathies lead to activation of proliferation-associated genes via derepression of WNT/β-catenin in various proliferative cell types (52–54). CHD8, connecting BAF and COMPASS complexes, negatively regulates β-catenin-targeted gene expression important in cellular proliferation and differentiation (55,56). In *Chd8* depleted mice, PRC2 regulation of WNT signaling appears to be the major altered pathway (57), and accordingly, an overgrowth phenotype with macrocephaly has been reported in CHD8-related NDD patients and animal models (28,58–60).

### Transcriptional convergence at the synapse in acute neurophysiological processes

Communication between the synapse and the nucleus in both directions is critical for neuronal development and plasticity. Synaptic activity triggers Ca-dependent signaling via signaling pathways such as PI3K-AKT-mTOR and RAS-MAPK that lead to activation and repression of genes that control neural connectivity and function. Integration of these activity-dependent transcriptional processes is mediated by some key transcription factors, such as CREB, MEF2 and MECP2, and heavily involves the regulation of chromatin organization. Well-known direct targets of these transcriptional complexes include immediate early genes such as *c-FOS*, *ARC* and *BDNF,* and disrupted expression of these genes is seen in many chromatinopathies (37,61–64). The deregulated expression appears to be a direct consequence of the causative mutation in these genes, and not just a secondary effect of neuronal pathology. Genome-wide expression studies in animal and cell models of chromatinopathies show an enrichment of differentially expressed genes that are associated with synaptic processes (65–69). For example, direct EHMT1 targets that were inappropriately repressed in the *Ehmt1^+/−^* mouse model for KS were linked to ion channels, synaptic vesicle and calcium signaling, and include *BDNF* as an important factor in the disrupted homeostatic plasticity in these animals (64,70–72). Epigenetic repressors such as EHMT1 and KDM5C interact with the RE1 silencing transcription factor (REST) to repress neuronal genes in extraneuronal tissues, and accordingly, loss of REST activity leads to upregulation of synaptic proteins, including SNAP25, Synapsin1, SCN2A, NMDAR1 and BDNF (73–75). In addition, EHMT1 appears to be a critical regulator of neuronal specification by regulation of protocadherins, possibly in conjunction with REST, and olfactory receptors.

Recent work using neuronal knockdown of genes from the KSS epigenetic module (EHMT1, MBD5, KMT2C and SMARCB1) identified commonly dysregulated classes of target genes, mainly involved in regulating neuronal excitability and synaptic function (76). However, the deregulation of individual genes from these classes shows differences in line with the specific activities of these proteins. Thus, knockdown of *KMT2C* and *SMARCB1* generally leads to downregulation of genes, whereas *EHMT1* knockdown yielded more upregulated than downregulated genes, including genes encoding proteins required for ion transport and synaptic transmission. *MBD5* knockdown had more subtle effects on the expression of these genes and in addition showed a more pronounced deregulation of cell adhesion and nucleosome assembly proteins. Thus, KSS target genes converge on neuronal excitability and synaptic function, albeit in variable patterns for each specific KSS gene. Despite these differences, the cumulative effect of deregulation of the synapse-related genes appears to result in a remarkably similar disruption of intrinsic synaptic properties and neuronal network activity.

## Neurophysiological phenotype convergence

An emerging concept of a common neurophysiological mode-of-failure is the excitation/inhibition (E/I) imbalance hypothesis, brought forward by Rubenstein and Merzenich in 2003, which postulates that excessive excitatory activity relative to inhibitory ones is a major contributor to autism pathology. This hypothesis was supported by the high comorbidity of epilepsy and autism (77). The E/I imbalance hypothesis has since grown into a broader concept encompassing a large range of perturbations within the excitation or inhibition neuronal circuits for a broader group of NDDs (78–80). E/I imbalance might be caused by alterations in glutamatergic excitatory drive, inhibitory connectivity or a combination thereof. As mentioned, KSS gene-deficient neuronal networks showed increased neuronal excitability, hyperactive networks with altered network organization and E/I balance (76). For all KSS genes, the shift in E/I balance was toward excitation. For EHMT1, this was associated with reduced number of inhibitory synapses, whereas for SMARCB1, KMT2C and MBD5, a reduction of both inhibitory and excitatory synapses was seen (76).

In Rett syndrome (RTT), the E/I balance can either be shifted toward excessive excitation or inhibition depending on the brain area. The importance of a tightly regulated E/I balance is demonstrated by the observation that knockout of *Mecp2* show elevated parvalbumin (PV) expression together with excessive excitatory inputs converging onto PV-expressing inhibitory neurons in the somatosensory and motor cortex associated with the sensorimotor defects (81). Distinct types of inhibitory neurons appear to contribute differently to phenotypic manifestations. Loss of MECP2 in PV^+^ neurons gives rise to progressive motor, sensory, memory and social deficits, whereas those lacking MeCP2 in somatostatin-positive (SOM^+^) neurons leads to seizures and stereotypic behavior. A similar observation was made for *Arid1b* haploinsufficiency, which in PV^+^ neurons contributed to social and emotional impairments, whereas Mecp2 loss in SOM^+^ neurons caused stereotypies as well as learning and memory dysfunction (54,82). An interesting observation is that the overall network imbalance contributing to the phenotypic regression in RTT involves *N*-methyl-*D*-aspartate receptor (NMDAR) dysfunction. Indeed, genetic deletion of the NMDAR subunit GluN2A in mice or NMDAR inhibition by ketamine prevented RTT phenotypes (83,84). Similarly, EHMT1 deficiency in both mice- and human-induced pluripotent stem cells-derived cortical excitatory neurons reduced the deposition of repressive H3K9me2 marks at the *GRIN1* promoter and, encoding an NMDAR subunit, leading to increased *GRIN1* expression and altered NMDAR function (85). Based on these preclinical observations, modulation of NMDAR is hypothesized to be a potential symptomatic treatment for several chromatinopathies, and clinical trials with ketamine are currently ongoing for RTT and Helsmoortel–Van der Aa syndrome.

**Figure 2:**
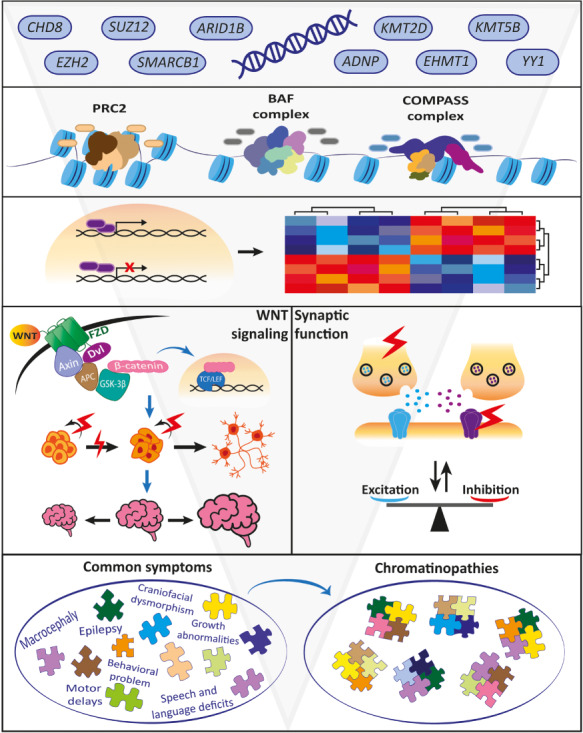
Schematic representation of convergence of chromatinopathies. Individual chromatinopathy genes encode proteins that interact with each other in chromatin remodeling. These interactions cluster in chromatin remodeling complexes such as the BAF, PRC2 and COMPASS complexes, which then affect the transcription of their target genes. Aberrant transcription level of various genes in different chromatinopathies leads to commonly disrupted molecular and cellular processes, such as WNT signaling and genes involved in acute synaptic function. Disrupted WNT signaling affects proliferation of stem cells and the process of neurogenesis, which can lead to anomalies in brain development such as micro- and macrocephaly. Synaptic function and activity is an important process in brain circuits development. Imbalance of excitation and inhibitory (E/I) has emerged as a common neurophysiological mode-of-failure in ASD, ID and NDDs in general. Combination of disruption of multiple developmental processes leads to commonly shared symptoms, which in different combinations and levels of severity builds onto specific chromatinopathy syndromes and disorders.

## Convergence as a target for therapy

Each of the individual chromatinopathy disorders has an extremely low prevalence, and therefore these disorders are not very attractive for developing gene-based interventions. However, the patterns of convergence that we have described for chromatinopathies might offer opportunities for developing therapeutic strategies that are applicable to a wider group of disorders. Such interventions can be directed at any level in the convergence pyramid (Fig. 2): protein complexes, gene expression, signaling pathways and cellular processes such as synaptic activity. This idea is not restricted to chromatinopathies and convergence can be seen for other NDDs as well. For example, disrupted mTor signaling is well known to be affected in a large number of different NDDs and is downstream of major signaling pathways, PI3K-AKT and RAS-MAPK, which are often directly affected by genetic variants in many rare syndromes (86). Consequently, rapamycin and other mTor modulators are being used in trials for ID and ASD disorders. Similarly, regulators of E/I balance and NMDAR antagonist like ketamine might be considered for treatment of several chromatinopathies. The downside of such generic interventions is that the expected effects are likely restricted to specific symptomatic features. Yet, very often these features are comorbid across a large number of NDDs, regardless of the classical categorical classification such as ID, ASD, schizophrenia and others. Therefore, such symptomatic interventions might be attractive to consider for many genetically distinct disorders. It is of eminent importance to further strengthen the patterns of convergence to increase knowledge about the underlying biological mechanisms in order to develop rational strategies for intervention applicable to larger groups of disorders caused by different genetic defects.

## Supplementary Material

Supplementary_Table_1_ddaa175

Supplementary_Table_2_ddaa175

Supplementary_Table_3_ddaa175
