# Supplementary material for: The phenomenal epigenome in neurodevelopmental disorders
Source: Hum Mol Genet. 2020 Aug 7;29(R1):R42–50. doi: 10.1093/hmg/ddaa175 (PMC7530535; doi:10.1093/hmg/ddaa175)
Supplement: Supplementary_Table_1_ddaa175 [file supplementary_table_1_ddaa175.docx]

| **Supplementary table 1: The child terms of and their relation to the GO term chromatin organization** |
| --- |
| **Child terms** |
| - positive regulation of chromatin organization (GO:1905269) - chromatin assembly or disassembly (GO:0006333) - nucleosome organization (GO:0034728) - chromatin remodeling (GO:0006338) - chromatin organization involved in negative regulation of transcription (GO:0097549) - sperm chromatin decondensation (GO:0035041) - sperm chromatin condensation (GO:0035092) - negative regulation of chromatin organization (GO:1905268) - heterochromatin organization (GO:0070828) - CENP-A containing chromatin organization (GO:0061641) - progressive alteration of chromatin involved in cell aging (GO:0001301) - covalent chromatin modification (GO:0016569) - nucleolar chromatin organization (GO:1990700) - chromatin organization involved in regulation of transcription (GO:0034401) - chromatin maintenance (GO:0070827) - regulation of chromatin organization (GO:1902275) - gene looping (GO:0090202) - chromatin silencing (GO:0006342) |
