# Supplementary material for: The phenomenal epigenome in neurodevelopmental disorders
Source: Hum Mol Genet. 2020 Aug 7;29(R1):R42–50. doi: 10.1093/hmg/ddaa175 (PMC7530535; doi:10.1093/hmg/ddaa175)
Supplement: Supplementary_Table_3_ddaa175 [file supplementary_table_3_ddaa175.docx]

**Supplementary Table 3:** Chromatinopathy genes There are 130 genes in total divided over categories. Writers (n = 31). 2: Erasers (n = 11). Remodellers: Superfamily II helicases (n = 21), Histones (n = 2), others readers and remodellers (n=66). The “Gene symbol” column contains the HGCN symbol (HUGO Gene Nomenclature Committee) of a gene. The “OMIM gene” column contains the OMIM id of a gene. The “function (epigenetics)” column contains the epigenetic function of a gene. The “Disease category” column describes whether a gene causes ID, autism spectrum disorder (ASD), ADHD, multiple congenital anomalies (MCA) and other features. The “OMIM disease” column contains the OMIM id of a disease. The “Inheritance” column describes the inheritance pattern of a gene. The “mutation details” column provides extra information about the type of mutation in the gene that causes the associated disease. AD, autosomal dominant; AR, autosomal recessive; XL, X-linked; LoF, loss of function; M missense; Fs, frameshift; Del, deletion; Dn, Dominant negative; GoF, gain of function. The origin colum provides Pubmed identifiers for those conditions that do not have an OMIM disease identifier.

| **Gene symbol** | **OMIMgene** | **Function (chromatin)** | **Disease category** | **Disease** | **OMIM disease** | **Inheritance** | **Mutation details** | **Origin** |
| --- | --- | --- | --- | --- | --- | --- | --- | --- |
| **Histone modifications: Writers** | | | | | | | | |
| **Methylation** | | | | | | | | |
| ASH1L | 607999 | Histone methyltransferase (H3K36me) | ID, ASD | Mental retardation, autosomal dominant 52 | 617796 | AD | LoF, M |  |
| EHMT1 / KMT1D | 607001 | Histone methyltransferase (H3K9me2/3) | ID, ASD, MCA | Kleefstra syndrome 1 | 610253 | AD | LoF |  |
| EZH2 / KMT6 | 601573 | Histone methyltransferase (H3K27me1/2/3) | ID | Weaver syndrome | 277590 | AD | M |  |
| KMT2A / MLL1 | 159555 | Histone methyltransferase (H3K4me) | ID, ASD, speech delay, MCA | Wiedemann-Steiner syndrome | 605130 | AD | LoF |  |
| KMT2B / MLL4 | 606834 | Histone methyltransferase (H3K4me) | Mild ID, growth retardation, dystonia | Dystonia 28, childhood-onset  Kleestra-like severe ID | 617284 | AD  AR | LoF  M | PMID: 25405613 |
| KMT2C / MLL3 | 606833 | Histone methyltransferase (H3K4me) | ID, ASD, MCA | Kleefstra-like syndrome (Kleefstra syndrome 2) | 617768 | AD | LoF |  |
| KMT2D / MLL2 | 602113 | Histone methyltransferase (H3K4me) | ID, growth retardation, microcephaly, MCA | Kabuki syndrome 1 | 147920 | AD | LoF |  |
| KMT2E / MLL5 | 608444 | Histone methyltransferase (H3K4me1/2) | ID, macrocephaly, MCA | O'Donnell-Luria-Rodan syndrome | 618512 | AD | LoF |  |
| KMT5B | 610881 | Histone methyltransferase (H4K20me3) | ID, Speech delay, tall | Mental retardation, autosomal dominant 51 | 617788 | AD | LoF |  |
| NSD1 / KMT3B | 606681 | Histone methyltransferase (H3K36, H4K20) | ID, overgrowth, macrocephaly, MCA | Sotos syndrome 1 | 117550 | AD | LoF, M |  |
| PRMT7 | 610087 | Histone methyltransferase (H4R3me2) | ID, short stature, MCA | Short stature, brachydactyly, intellectual developmental disability, and seizures | 617157 | AR | LoF |  |
| SETD1A / KMT2F | 611052 | Histone methyltransferase (H3K4me) | Schizophrenia, Epilepsy, structural brain anomalies | Epilepsy, early-onset, with or without developmental delay | 618832 | AD | LoF, M |  |
| SETD1B / KMT1G | 604396 | Histone methyltransferase (H3K4me3) | ASD, epilepsy, ID | mild to profound ID, epilepsy and autistic behaviour | - | AD | LoF, M | PMID:  28135719, 31110234, 29322246, 31440728 |
| SETD2 / KMT3A | 612778 | Histone methyltransferase (H3K36me3) | ID, ASD, speech delay, behavioural | Luscan-Lumish syndrome | 616831 | AD | LoF |  |
| SETD5 | 615743 | Histone methyltransferase (H3K36me1/3) | ID, ASD, delayed speech | Mental retardation, autosomal dominant 23 | 615761 | AD | LoF |  |
| WHSC1 / NSD2 | 602952 | Histone methyltransferase (H3K36me3, H3K27me, H4K20) | ID, DD, microcephaly | developmental delay, ID, microcephaly, short stature, facial dysmorphism, hypotonia, teeth anomalies | - | AD | Del, LoF | PMID:  29892088, 29760529, 30345613 |
| **Acetylation** | | | | | | | | |
| CREBBP | 600140 | Histone acetyltransferase | ID + S. autism | Rubinsteun-Taybi syndrome  Menke-Hennekam syndrome 1 | 180849  618332 | AD | LoF, M |  |
| EP300 | 602700 | Histone acetyltransferase (H3K122ac, H3K27ac), Histone crotonyltransferase, Histone butyryltransferase, | ID + S. autism | Rubinstein-Taybi syndrome 2  Menke-Hennekam syndrome 2 | 613684  618333 | AD  AD | LoF, M  M |  |
| KAT6A | 601408 | Histone acetyltransferase (H3, H4 | ID, microcephaly, speech delay, MCA | Arboleda-Tham syndrome | 616268 | AD | LoF |  |
| KAT6B | 605880 | Histone acetyltransferase | ID, micro-cephaly, MCA | Genitopatellar syndrome  SBBYSS syndrome | 606170  603736 | AD  AD | LoF  -D |  |
| TAF1 | 313650 | Histone acetyltransferase (H3, H4) | ID, ASD, growth retardation, MCA | Mental retardation, X-linked, syndromic 33  Dystonia-Parkinsonism, X-linked | 300966  314250 | XL  XL | M  INS |  |
| TAF6 | 607798 | Part of PCAF histone acetylase complex | ID, short stature, microcephaly | Alazami-Yuan syndrome | 617126 | AR | M |  |
| **Phosphorylation** | | | | | | | | |
| VRK1 | 602168 | Histone kinase (H3) | ID | Pontocerebellar hypoplasia type 1A | 607596 | AR | LoF, M |  |
| **Ubiquitination** | | | | | | | | |
| HUWE1 | 300697 | Histone ubiquitin ligase (H2AK119Ub) | ID, ASD, growth retardation | Mental retardation, X-linked syndromic, Turner type | 300706 | XL | M/iF |  |
| TRIM37 | 605073 | Histone ubiquitin ligase (H2AK119Ub) |  | MULIBREY NANISM | 213300 | AR | LoF |  |
| TRIP12 | 604506 | E3 ubiquitin-protein ligase (H2AK63Ub) | ID, ASD | Mental retardation, autosomal dominant 49 | 617752 | AD | LoF |  |
| UBE2A | 312180 | monoubiquitination of H2BK120ub1 | ID, MCA | Mental retardation, X-linked syndromic, Nascimento-type | 300860 | XL | LoF, M |  |
| **Biotinylation** | | | | | | | | |
| HLCS | 609018 | Histone biotin ligase | ID | Holocarboxylase synthetase deficiency | 253270 | AR | LoF |  |
| **O-GlcNAcylation** | | | | | | | | |
| OGT | 300255 | Histone O-GlcNAcyltransferase (H2BS112O-GlcNAc), NSL complex | ID, microcephaly | Mental retardation, X-linked 106 | 300997 | XL | M, SS |  |
| **DNA modifications: Writers** | | | | | | | | |
| DNMT3A | 602769 | DNA methyltransferase | ID, growth defects | Tatton-Brown-Rahman syndrome  Heyn-Sproul-Jackson syndrome | 615879  618724 | AD  AD | LoF  GoF |  |
| DNMT3B | 602900 | DNA methyltransferase, Involved in histone methylation | ID | Immunodeficiency-centromeric instability-facial anomalies syndrome 1 | 242860 | AR | LoF |  |
| **Histone modifications: Erasers** | | | | | | | | |
| **Demethylation** | | | | | | | | |
| KDM1A /  LSD1 | 609132 | Histone demethylase (H3K4me, H3K9me) | ID | Cleft palate, psychomotor retardation, and distinctive facial features | 616728 | AD | M |  |
| KDM3B | 609373 | Histone demethylase (H3K9me3) | ID, speech delay, short stature, MCA | Diets-Jongmans syndrome | 618846 | AD | M, LoF |  |
| KDM5B / JARID1B | 605393 | Histone demethylase (H3K4me1/2/3) | ID, delayed speech, MCA | Mental retardation, autosomal recessive 65 | 618109 | AR | LoF |  |
| KDM5C/ SMCX /JARID1C | 314690 | Histone demethylase (H3K4me2/3) | ID + S. autism | Mental retardation, X-linked, syndromic, Claes-Jensen type | 300534 | XL | LoF, M |  |
| KDM6A / UTX | 300128 | Histone demethylase (H3K27me2/3) | ID, reduced growth, MCA | Kabuki syndrome 2 | 300867 | XL | LoF |  |
| KDM6B | 611577 | Histone demethylase (H3K27me2/3) | ID, speech apraxia, MCA | Neurodevelopmental disorder with coarse facies and distal skeletal abnormalities | 618505 | AD | LoF, M |  |
| PHF8 | 300560 | Histone demethylase (H3K9me1/2, H3K27me2, H4K20me1) | ID. MCA | Mental retardation syndromic x-linked siderius type | 300263 | XL | LoF |  |
| **Deacetylation** | | | | | | | | |
| HDAC4 | 605314 | Histone deacetylase | ID | Brachydactyly-mental retardation syndrome | 600430 | AD | LoF |  |
| HDAC8 | 300269 | Histone deacetylase, Involved in telomere maintenance | ID | Cornelia de Lange syndrome 5 | 300882  309585 | XL | M, LoF |  |
| **Deubiquitination** | | | | | | | | |
| USP7 | 602519 | Histone deubiquitination (H2B). Involved in DNA methylation | ID, ASD | intellectual disability, autism spectrum disorder, epilepsy, aggressive behavior, hypotonia, and hypogonadism | - | AD | Del, LoF | PMID:  26365382, 30679821 |
| **DNA modifications: Erasers** | | | | | | | | |
| TET3 | 613555 | catalyzes the conversion of 5-methylcytosine (5mC) into 5-hydroxymethylcytosine (5hmC) | ID, ASD, ADHD, growth defects, MCA | Beck-Fahrner syndrome | 618798 | AD, AR | LoF,  M |  |
| **Superfamily II helicases (Remodellers)** | | | | | | | | |
| ATRX | 300032 | Helicase, ATRX:DAXX complex: Chromatin remodeling, Telomere maintenance, Involved in histone methylation | ID + S. autism | -Mental retardation syndromic x-linked with hypotonic facies syndrome type 1 -alpha-thalassemia mental retardation syndrome x-linked non-deletion type | 309580 301040 | XL  XL | LoF  LoF |  |
| **CHD subfamily** | | | | | | | | |
| CHD1 | 602118 | ubiquitously expressed ATP-dependent chromatin remodeling protein | ID, DD, seizures, macrocephaly, speech apraxia | Pilarowski-Bjornsson syndrome | 617682 | AD | M/iF |  |
| CHD2 | 602119 | Helicase | ID, ASD | Epileptic encephalopathy, childhood-onset | 615369 | AD | MG CNV |  |
| CHD3 | 602120 | Helicase, NuRD complex: Histone deacetylation | ID, speech apraxia, macrocephaly | Snijders Blok-Campeau syndrome | 618205 | AD | M/iF |  |
| CHD4 | 603277 | Helicase, NuRD complex: Histone deacetylation | ID, heart defects | Sifrim-Hitz-Weiss syndrome | 617159 | -AD -AD | M/iF |  |
| CHD7 | 608892 | Helicase, | Syndromic ID | Hypogonadotropic hypogonadism 5 with or without anosmia  Charge syndrome | 612370  214800 | AD | LoF |  |
| CHD8 | 610528 | Helicase | ASD, ID, macrocephaly | Autism, susceptibility to, 18 | 615032 | AD | LoF |  |
| **INO80/SWR1 complex** | | | | | | | | |
| SRCAP | 611421 | Catalytic component of the SRCAP complex mediating ATP-dependent remodeling. Acts as a coactivator for CREB-mediated transcription. | ID, short stature, MCA | Floating-Harbor syndrome | 136140 | AD | DN |  |
| **ISWI complex** | | | | | | | | |
| BPTF | 601819 | bromodomain PHD finger transcription factor .Histone-binding component of NURF. ISWI complex | ID | Neurodevelopmental disorder with dysmorphic facies and distal limb anomalies | 617755 | AD | LoF, M |  |
| **SWI/SNF complex** | | | | | | | | |
| ACTL6A | 604958 | BRG1-Associated Factor, BAF53A, | ID | Coffin-Siris like syndrome |  | AD | LoF, M | PMID:  28649782, 31994175 |
| ACTL6B | 612458 | BRG1-Associated Factor, BAF53B | ID, epilepsy | ID with severe speech and ambulation defects  Epileptic encephalopathy | 618470  618468 | AD  AR | M/iF  LoF |  |
| ARID1A | 603024 | SWI/SNF chromatin remodeling complexes: BAF250A, SMARCF1 | ID, MCA | Coffin-Siris syndrome 2 | 614607 | AD | LoF |  |
| ARID1B | 614556 | SWI/SNF chromatin remodeling complexes: BAF250B | ID, MCA | Coffin-Siris syndrome 1 | 135900 | AD | LoF |  |
| ARID2 | 609539 | SWI/SNF-B (PBAF) chromatin remodeling complex: BAF200 | ID, MCA | Coffin-Siris syndrome 6 | 617808 | AD | LoF |  |
| DPF2 | 601671 | SWI/SNF-B (PBAF) chromatin remodeling complex | ID, MCA | Coffin-Siris syndrome 7 | 618027 | AD | LoF, M |  |
| SMARCA2 | 600014 | Helicase, SNF2L2 | ID, MCA | Nicolaides-Baraitser syndrome | 601358 | AD | M, LoF |  |
| SMARCA4./.BRG1 | 603254 | Helicase, Involved in histone deacetylation | ID, ASD, MCA | Coffin-Siris syndrome 4  Rhabdoid tumor predisposition syndrome 2 | 614609  613325 |  | M  LoF |  |
| SMARCB1./ SNF5 | 601607 | BAF complex: chromatin remodeling, Involved in histone acetylation, methylation | ID, ASD, MCA | Coffin-Siris syndrome 3  Kleefstra-like syndrome  Rhabdoid tumor predisposition syndrome 2 | 614608  609322 | AD AD SOM | M  M  LoF |  |
| SMARCC2 | 601734 | SWI/SNF chromatin remodeling complexes: BAF170 | ID, MCA | Coffin-Siris syndrome 8 | 618362 | AD | M, LoF |  |
| SMARCD1 | 601735 | BAF60A subunit of the SWI/SNF chromatin remodeling complex | ID, MCA | Coffin-Siris syndrome 11 | 618779 | AD | M, LoF |  |
| SMARCE1 | 603111 | BAF57 subunit of the SWI/SNF chromatin remodeling complex | ID, MCA | Coffin-Siris syndrome 5  Familial meningioma | 616938  607174 | AD  AD | M  LoF |  |
| **Histones** | | | | | | | | |
| HIST1H1E | 142220 | Histone | ID, DD, increases height/weight | Rahman syndrome | 617537 | AD | LoF |  |
| HIST1H4C | 602827 | Histone | ID, growth delay, MCA | ID, growth retardation, microcephaly, hypotonia, facial dysmorphism, foot ray anomaly | - | AD | M | PMID: 28920961 |
| **Other (readers, remodellers)** | | | | | | | | |
| ACTB | 102630 | NuA4 histone acetyltransferase complex: Histone acetylation, Involved in chromatin remodeling | ID | Baraitser-Winter syndrome 1 Dystonia, juvenile-onset | 243310 607371 | AD AD | M  M |  |
| ADNP | 611386 | Activity-dependent neuroprotector homeobox protein. Interacts with BAF complex and β-catenin | Syndromic ASD | Helsmoortel-Van der Aa Syndrome | 615873 | AD | LoF |  |
| ALG13 | 300776 | Putative bifunctional UDP-N-acetylglucosamine transferase and deubiquitinase | Epilepsy, ID | Congenital disorder of glycosylation  Epileptic encephalopathy | 300884 | XL | M |  |
| ASXL1 | 612990 | PR-DUB complex: Histone deubiquitination (H2AK119ub1), Putative Polycomb group (PcG) protein | ID | Bohring-Opitz syndrome | 605039 | AD | LoF |  |
| ASXL2 | 612991 | PR-DUB complex: Histone deubiquitination (H2AK119ub1), Putative Polycomb group (PcG) protein | ID | Shashi-Pena syndrome | 617190 | AD | LoF |  |
| ASXL3 | 615115 | PR-DUB complex: Histone deubiquitination (H2AK119ub1), Putative Polycomb group (PcG) protein | ID | Bainbridge-Ropers syndrome | 615485 | AD | LoF |  |
| AUTS2 | 607270 | Component of Polycomb PRC1-like complex | ID, ASD | Mental retardation, autosomal dominant 26 | 615834 | AD | LoF |  |
| BCOR | 300485 | Involved in histone ubiquitination (H2A), Involved in histone methylation, Involved in histone acetylation | ID | Microphthalmia syndromic type 2 | 300166 | XL | LoF |  |
| BCORL1 | 300688 | Involved in histone acetylation/deacetylation | ID | Shukla-Vernon syndrome | 301029 | XL | M |  |
| BRD4 | 608749 | Bromodomain-containing protein; Chromatin reader protein that recognizes and binds acetylated histones | ID, ASD | Cornelia de Lange-like syndrome | - | AD | LoF, M | PMID:  29379197 |
| BRPF1 | 602410 | MOZ/MORF complex: Histone acetylation (H3K23ac) | ID | -BRPF1 associated syndromic intellectual disability with ptosis | 617333 | AD | LoF, M |  |
| BRWD3 | 300553 | Bromodomain- and wd repeat-containing protein | ID | Mental retardation, X-linked 93 | 300659 | XL | LoF, M |  |
| CDK9 | 603251 | Modulates chromatin modifications and integrates phosphorylation with chromatin modifications | ID, DD, short stature | moderate ID, short stature; developmental delay, epilepsy, MRI anomalies and MCA | - | AR | M,iF | PMID:  29302074, 30237576 |
| CTBP1 | 602618 | Involved in histone acetylation | ID | Hypotonia, ataxia, developmental delay, and tooth enamel defect syndrome | 617915 | AD | M |  |
| CTCF | 604167 | Chromatin insulator, Involved in DNA methylation, Involved in histone acetylation, Involved in histone methylation | ID | Mental retardation, autosomal dominant 21 | 615502 | AD | LoF |  |
| CTNNB1 | 116806 | Involved in histone methylation, Involved in telomere maintenance via telomerase | ID, ASD | Mental retardation, autosomal dominant 19 | 615075 | AD | LoF |  |
| CUL4B | 300304 | E3 ubiquitin-protein ligase complex: Histone ubiquitination | ID | Mental retardation syndromic x-linked cabezas type | 300354 | XL | LoF |  |
| DDB2 | 600811 | DDB1-CUL4-ROC1 complex: Histone ubiquitination (H2A, H3, H4) |  | Xeroderma pigmentosum, group e, ddb-negative subtype | 278740 | AR | LoF |  |
| DDX11 | 601150 | DEAD box helicase family (DEAH subfamily) | ID | Warsaw breakage syndrome | 613398 | AR | LoF |  |
| EED | 605984 | PRC2/EED-EZH2 complex: Histone methylation (H3K9me H3K27me), May regulate DNA methylation | ID | Cohen-Gibson syndrome | 617561 | AD | M |  |
| ERCC6 | 609413 | DNA double-strand breaks (DSBs) repair pathway and G2/M checkpoint activation; DNA-dependent ATPase activity | ID, dwarfism, MCA | Cockayne syndrome, type B and various other syndromes | 133540 | AR | LoF |  |
| FMR1 | 309550 | Involved in histone phosphorylation | ID, ASD, MCAG | Fragile X syndrome  Fragile X tremor/ataxia syndrome  Premature ovarian failure 1 | 300624  300623  311360 | XL | Repeat |  |
| GATAD2B | 614998 | Involved in chromatin remodeling, MeCP1 histone deacetylase complex: Histone deacetylation | ID | Mental retardation, autosomal dominant 18 | 615074 | AD | LoF |  |
| GPX4 | 138322 | Anti-oxidant properties. paternal chromatin decondensation and male pronucleus formation at fertilization | Structural brain defects, growth retardation | Spondylometaphyseal dysplasia, Sedaghatian type | 250220 | AR | LoF |  |
| HCFC1 | 300019 | Involved in histone acetylation, Involved in histone deacetylation, Involved in histone methylation | ID, ASD | Mental retardation, X-linked 3 –(Cobalbumin disorder) | 309541 | XL | M, LoF |  |
| HNRNPU | 602869 | Involved in telomere maintenance | ID, ASD | Epileptic encephalopathy, early infantile, 54 | 617391 | AD | LoF |  |
| KANSL1 | 612452 | Kat8 regulatory NSL complex, subunit 1: involved in Histone acetylation (H4) | ID | Koolen-De Vries syndrome | 610443 | AD | LoF |  |
| KPNA7 | 614107 | Importin-alpha family of karyopherins. Positive regulation of histone H3-K27 trimethylation | ID, epilepsy, growth retardation, microcephaly | severe developmental delay, epilepsy, ACC, cerebellar vermis hypoplasia; plus nonsense variant in 2 sibs with moderate ID, short stature, mild microcephaly and seizures, normal MRI | - | AD | M | PMID:  24045845, 29302074 |
| MAP3K7 | 602614 | Involved in histone acetylation | ID | Frontometaphyseal dysplasia 2  Cardiospondylocarpofacial syndrome-intellectual disability | 617137 157800  No MIM | AD AD AR | M  M, Del  M | PMID:  28397838 |
| MBD5 | 611472 | Binds to heterochromatin, but does not interact directly with either methylated or unmethylated DNA (in vitro) | ID, short stature, MCA | Mental retardation, autosomal dominant 1 (Smith-Magenis-like)  Kleefstra-like syndrome | 156200 | AD | Del, LoF | PubMed: 22726846 |
| MCM3AP | 603294 | Through the acetylation of histones, affects the assembly of nucleosomes | ID, speech delay, neuropathy | Peripheral neuropathy, autosomal recessive, with or without impaired intellectual development | 618124 | AR | LoF, M |  |
| MECP2 | 300005 | Binds both 5-methylcytosine (5mC) and 5-hydroxymethylcytosine (5hmC)-containing DNA. Acts either as repressor or activator. Mediates transcriptional repression through interaction with histone deacetylase and the corepressor SIN3A. | ID, ASD, MCA, neuro-degeneration | Rett syndrome (RTT)  Mental retardation syndromic x-linked lubs type  Mental retardation syndromic x-linked type 13  encephalopathy neonatal severe  Autism susceptibility | 312750 300260  300055  300673 300496 | -XL -XL -XL -XL -XL -XL | LoF Dup  M  M  PS | Premature stip |
| MORC2 | 616661 | ATPase essential for epigenetic silencing by the HUSH (human silencing hub) complex | Peripheral neuropathy, Learning disability | Charcot-Marie-Tooth disease, axonal, type 2Z | 616688 | AD | M |  |
| MSL3 | 300609 | MSL complex: Histone acetylation (H4K16ac), Involved in chromatin remodeling | ID | Basilicata-Akhtar syndrome | 301032 | XL | LoF |  |
| NCOR1 | 600849 | large corepressor complex that contains SIN3A/B and histone deacetylases HDAC1 and HDAC2: Histone deacetylation | ASD, syndromic | ASD, bifid uvula and early-onset scoliosis | - | AD | M, SS | PMID:  27824329, 30289594 |
| NIPBL | 608667 | Involved in histone deacetylation | ID, growth retardation, MCA | Cornelia de Lange syndrome 1 | 122470 | AD | LoF, M |  |
| ORC1 | 601902 | omponent of the origin recognition complex (ORC) that binds origins of replication. DNA-binding is ATP-dependent. | ID, reduced height, weight, MCA | Meier-Gorlin syndrome 1 | 224690 | AR | LoF, M |  |
| PAK1 | 602590 | phosphorylates MORC2 which activates its ATPase activity and facilitates chromatin remodeling | ID, macrocephaly, seizures, MCA | Intellectual developmental disorder with macrocephaly, seizures, and speech delay | 618158 | AD | GoF |  |
| PCGF2 | 600346 | PRC1-like complex: Histone ubiqutination (H2AK119ub1) | ID, structural brain anomalies | Turnpenny-Fry syndrome | 618371 | AD | M |  |
| PHF21A | 608325 | Part of BHC corepressor complex. Represses neuron-specific genes in non-neuronal cells upon recruitment by REST. Acts by deacetylating and demethylating histones | ID, ASD, ADHD, MCA | Intellectual developmental disorder with behavioral abnormalities and craniofacial dysmorphism with or without seizures | 618725 | AD | LoF |  |
| PHF6 | 300414 | Chromatin binding. Copurified with nucleosome remodeling and deacetylation (NuRD) complex proteins CHD4, HDAC1, and RBBP4. | ID, short stature, microcephaly, MCA | Borjeson-Forssman-Lehmann syndrome  Coffin-Siris like phenotype | 301900  - | XL | LoF, M  FS, M | PMID: 23906836 |
| PHIP | 612870 | Lysine-acetylated histone binding. Probable regulator of the insulin and insulin-like growth factor signaling pathways | ID, ASD, ADHD, obesity, MCA | Chung-Jansen syndrome | 617991 | AD | LoF, M |  |
| PPM1D | 605100 | Involved in chromatin silencing.  Phosphatase, a nucleolar localized protein, relocates to chromatin during replication stress | ID, ASD, OCD, short stature | Jansen de Vries syndrome  (Intellectual developmental disorder with gastrointestinal difficulties and high pain threshold) | 617450 | AD | LoF |  |
| RAI1 | 607642 | Regulates transcription through chromatin remodeling by interacting with other proteins in chromatin | ID, speech delay, MCA | Smith-Magenis syndrome | 182290 | AD | LoF, M |  |
| RBPJ | 147183 | DNA Methylation and Sequence-Specific Binding Protein | Psychomotor retardation, microcephaly | Adams-Oliver syndrome 3 | 614814 | AD | M |  |
| RCBTB1 | 607867 | Regulator of chromosome condensation and BTB domain-containing protein 1. Involved in chromatin remodeling | Mild ID | Retinal dystrophy with or without extraocular anomalies | 617175 | AR | M |  |
| RERE | 605226 | Involved in chromatin binding and remodeling | ASD, short stature | Neurodevelopmental disorder with or without anomalies of the brain, eye, or heart | 616975 | AD | LoF, M |  |
| SATB2 | 608148 | Acts as a docking site for several chromatin remodeling enzymes and also by recruiting corepressors (HDACs) or coactivators (HATs) directly to promoters and enhancers | ID, growth retardation, cleft palate, MCA | Glass syndrome | 612313 | AD | LoF, M |  |
| SET | 600960 | Multitasking protein, involved in apoptosis, transcription, nucleosome assembly and histone chaperoning.  Involved in histone acetylation | ID | Mental retardation, autosomal dominant 58 | 618106 | AD | LoF, M |  |
| SIN3A | 607776 | Involved in histone deacetylation with HDAC1 en HDAC2. Corepressor for REST. | ID, ASD, ADHD, growth retardation, MCA | Witteveen-Kolk syndrome | 613406 | AD | LoF |  |
| SKI | 164780 | Involved in histone acetylation. Stops, prevents or reduces the activity of histone deacetylase. | ID, microcephaly, MCA | Shprintzen-Goldberg craniosynostosis syndrome | 182212 | AD | M |  |
| SMAD4 | 600993 | Chromatin binding. Involved in histone methylation, Involved in histone acetylation. Acts synergistically with SMAD1 and YY1 | ID, ASD, short stature, MCA | Myhre syndrome Juvenile polyposis/hereditary hemorrhagic telangiectasia syndrome Juvenile polyposis syndrome  Pancreatic cancer | 139210 175050  174900  260350 | AD AD  AD  SOM | GoF LoF LoF  LoF  LoF |  |
| SOX2 | 184429 | Involved in chromatin organization | ID | Syndromic microphthalmia-3 (MCOPS3) | 206900 | AD | LoF, M |  |
| SUPT16H | 605012 | Component of the FACT (facilitates chromatin transcription) remodeling complex | ID, ASD, MCA | mild to severe ID, autistic features, dysmorphism, 2/5 seizures, corpus callosum anomalies and decreased white matter volume, | - | AD | LoF, M | PMID:  31924697 |
| SUZ12 | 606245 | Polycomb group (PcG) protein. Component of the PRC2/EED-EZH2 complex | ID, overgrowth | Imagawa-Matsumoto syndrome | 618786 | AD | LoF, M |  |
| TBL1XR1 | 608628 | Involved in histone deacetylation | IDTCF | Mental retardation, autosomal dominant 41  Pierpont syndrome | 616944  602342 | AD AD | M, FS  M |  |
| TCF20 | 603107 | Chromatin binding transcriptional coactivator | ID, ASD, ADHD, overgrowth, MCA | Developmental delay with variable intellectual impairment and behavioral abnormalities | 618430 | AD | LoF |  |
| TLK2 | 608439 | Serine/threonine-protein kinase involved in chromatin assembly | ID, ASD, ADHD, MCA | Mental retardation, autosomal dominant 57 | 618050 | AD | LoF |  |
| TRRAP | 603015 | NuA4 histone acetyltransferase complex: Histone acetylation, Involved in chromatin remodeling | ID, ASD, dysmorphism, MCA | Developmental delay with or without dysmorphic facies and autism  Deafness | 618454  618778 | AD  AD | M  M |  |
| TWIST1 | 601622 | Involved in histone acetylation, Involved in histone phosphorylation | -  -  -  ID | Saethre-Chotzen syndrome Craniosynostosis, type 1  Robinow-Sorauf syndrome  Sweeney-Cox syndrome | 101400 123100  180750  617746 | AD AD  AD  AD | LoF  M  LoF  M |  |
| WAC | 615049 | Involved in histone ubiquitination (H2BK120ub1) | ID, ASD, MCA | Desanto-Shinawi syndrome | 616708 | AD | LoF |  |
| YY1 | 600013 | Involved in chromatin looping. Recruits PRC2/EED-EZH2 to target genes and the INO80 complex to YY1-responsive elements | ID, ASD, MCA | Gabriele-de Vries syndrome | 617557 | AD | LoF, M |  |
| ZMIZ1 | 607159 | chromatin-mediated maintenance of transcription | ID, DD. MCA | NDD with dysmorphic facies and distal skeletal anomalies | 618659 | AD | Fs, M |  |
| ZMYND11 | 608668 | Chromatin reader (H3.3K36me3) | ID, DD, speech delay | Mental retardation, autosomal dominant 30 | 616083 | AD | LoF |  |
| ZNF335 | 610827 | Component of histone methyltransferase complexes. Links COMPASS and REST complexes | DD, structural brain anomalies, MCA | Microcephaly 10, primary, autosomal recessive | 615095 | AR | M |  |
| ZNF462 | 617371 | Involved in chromatin organization | ID, ASD, ADHD | Weiss-Kruszka syndrome | 618619 | AD | LoF |  |
